# A case of pituitary gland abscess associated with granulomatous hypophysitis

**DOI:** 10.1186/s12883-023-03060-6

**Published:** 2023-01-12

**Authors:** Mohammad Mahdi Rabiei, Kaveh Ebrahimzadeh, Zahra Davoudi, Farahnaz Bidari Zerehpoosh, Farid Javandoust Gharehbagh, Roghayeh Sedaghati, Legha Lotfollahi, Fatemeh Kalhor, Ilad Alavi Darazam

**Affiliations:** 1 Infectious Diseases and Tropical Medicine Research Center, Shahid Beheshti University of Medical Sciences, Tehran, Iran; 2 Department of Infectious Diseases and Tropical Medicine, Loghman Hakim Hospital, Shahid Beheshti University of Medical Sciences, Tehran, Iran; 3 Skull Base Research Center, Loghman Hakim Hospital, Shahid Beheshti University of Medical Sciences, Tehran, Iran; 4 Department of Endocrinology, Skull Base Research Center of Loghman Hakim Hospital, Shahid Beheshti University of Medical Sciences, Tehran, Iran; 5 Department of Pathology, Loghman Hakim Hospital, Shahid Beheshti University of Medical Sciences, Tehran, Iran; 6 Department of Nephrology, Loghman Hakim Hospital, Shahid Beheshti University of Medical Sciences, Tehran, Iran; 7 Shahid Beheshti University of Medical Sciences, Tehran, Iran

**Keywords:** Granulomatous hypophysitis, Abscess, Pituitary gland, Streptococcus viridans, Case report

## Abstract

**Background:**

Granulomatous hypophysitis is a rare disease that presents with chronic inflammation of the pituitary gland. In this study, we reported a case of granulomatous hypophysitis associated with a pituitary abscess.

**Case presentation:**

A 39-year-old woman presented with a 2-year history of infertility. For the past six months, she has suffered from amenorrhea, decreased libido, headaches, and vertigo. She was referred to our hospital with a suspected diagnosis of nonfunctioning pituitary adenoma based on her presentation and brain MRI findings. She underwent trans-sphenoidal surgery (TSS). Direct observation during surgery revealed drainage of malodor pus and pituitary gland abscess. The histopathological evaluation also showed granulomatous hypophysitis and neutrophilic microabscess formation.

The patient was initially treated with high doses of ceftriaxone (2 g twice daily) and metronidazole (500 mg (mg) four times per day). Also, the patient received cortisol replacement therapy after the operation. After obtaining the antibiogram and culture results, the treatment regimen was continued for 4 weeks postoperatively, followed by amoxicillin-clavulanate (500/125 mg three times daily) for a total duration of 12 weeks.

**Conclusion:**

The patient recovered uneventfully and the postoperative MRI was normal without any remnant lesions.

## Background

Hypophysitis is a rare disease caused by inflammatory infiltration and destruction of the pituitary gland [1]. The prevalence is unknown as only a few reports have been considered. Hypophysitis has five types: lymphocytic, granulomatous, xanthomatous, xanthogranulomatous, and necrotizing. Of all types, lymphocytic and granulomatous are the most common types [1–4]. Lymphocytic hypophysitis usually occurs in females and is diagnosed during pregnancy or postpartum, and the majority of patients develop anterior pituitary dysfunction [5]. Granulomatous hypophysitis is also divided into primary (idiopathic) and secondary, which can lead to tuberculosis, sarcoidosis, syphilis, Langerhans histiocytosis, granulomatosis with polyangiitis (formerly known as Wegener’s granulomatosis), and Rathke’s cleft cyst rupture [1, 2].

Most pituitary inflammatory lesions occur in women in the postpartum period. Symptoms such as headache, visual symptoms, and involvement of the pituitary gland and stalk may result in endocrine dysfunction, often resulting from the influence of a mass lesion within the sella [3, 4]. Hypophysitis can also cause hyperprolactinemia due to stalk disruption. Also, Amenorrhea can occur due to several multiple mechanisms [5].

A pituitary abscess is a rare and life-threatening purulent inflammatory disease that accounts for less than 1% of all pituitary lesions [6]. Clinical diagnosis is difficult because patients do not have specific clinical symptoms and it is often radiologically indistinguishable from other pituitary lesions [7].

In this study, we reported a case of granulomatous hypophysitis associated with a pituitary abscess.

### Case presentation

A 39-year-old Iranian woman presented with menstrual disturbance, headache, vertigo, decreased libido, polyuria, and polydipsia for six months. In addition, she has been infertile for two years. She was a housewife with no history of occupational exposure and her family history was unremarkable. She never smoked or drank alcohol. The patient denied a history of diabetes mellitus or hypertension, but she noted a head injury 4 years ago with no change in the level of consciousness and no evidence of skull base fracture or leakage. She did not suffer from hyperandrogenism, visual complaints, and diplopia. Moreover, no signs of acromegaly or Cushing syndrome were detected.

Vital signs on admission remained normal. Physical examination revealed no visual field defects, and fundoscopy showed that both eyes were normal with sharp optic disc margins. Her visual acuity was 10/10 bilaterally. All neurological examinations, including cranial nerves, were normal.

**Fig. 1:**
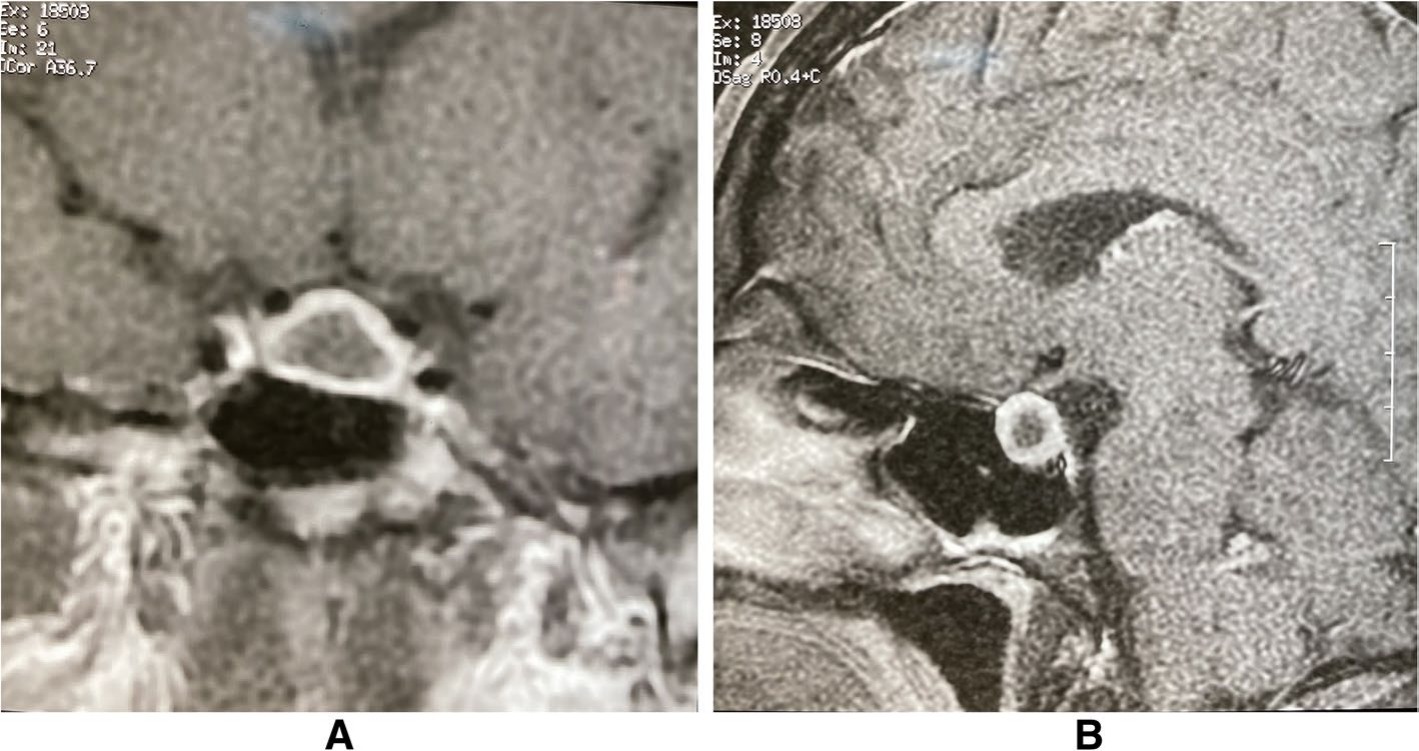
**A**, **B** Coronal (**A**) and Sagittal (**B**) post contrast T1-weighted images of pituitary gland demonstrate pituitary enlargement with rim enhancing hypointense lesion, superior pituitary convexity, and stalk thickening are also depicted

Further investigations at another center revealed elevated levels of prolactin (Table 1); therefore, a dynamic pituitary gland magnetic resonance imaging (MRI) of the pituitary was performed, showing an enlarged gland with a thick infandibulum and a peripherally enhancing T1 hypo intense lesion. (Fig. 1A, B).

**Table 1:** The primary laboratory investigations at the first clinical visit

Test	Measured value	Reference value	Unit
TSH	3.2	0.45–4.5	mU/ L
T4	6	5–12	micg/ dl
LH	1.7	Follicular phase: 1.37–9 Midcycle peak: 6.17–17.2 Luteal phase: 1.09–9.2	IU/ L
FSH	14	1.5–12.4	IU/ L
PRL	73	Non- pregnant: < 25	ng/ ml
PRL (diluted)	52	2.8–29.2	ng/ ml
Cortisol (8 A.M)	7.5	10–20	micg/ dl

*TSH* Thyroid Stimulating Hormone, *T4* Thyroxin, *LH* Luteinizing Hormone, *FSH* Follicular Stimulating Hormone, *PRL* Prolactin, *B-HCG* Beta Human Gonadotropic Hormone

According to the results of clinical and laboratory investigations, the patient underwent trans-sphenoidal surgery (TSS) with the primary diagnosis of non-functional pituitary macro-adenoma. However, direct observation during surgery revealed drainage of malodor pus when the sella was opened. The putrid collection was drained and the enlarged and inflamed part of the pituitary gland was excised. Cerebrospinal fluid leakage and sinus tracts were evaluated after surgery.

**Fig. 2:**
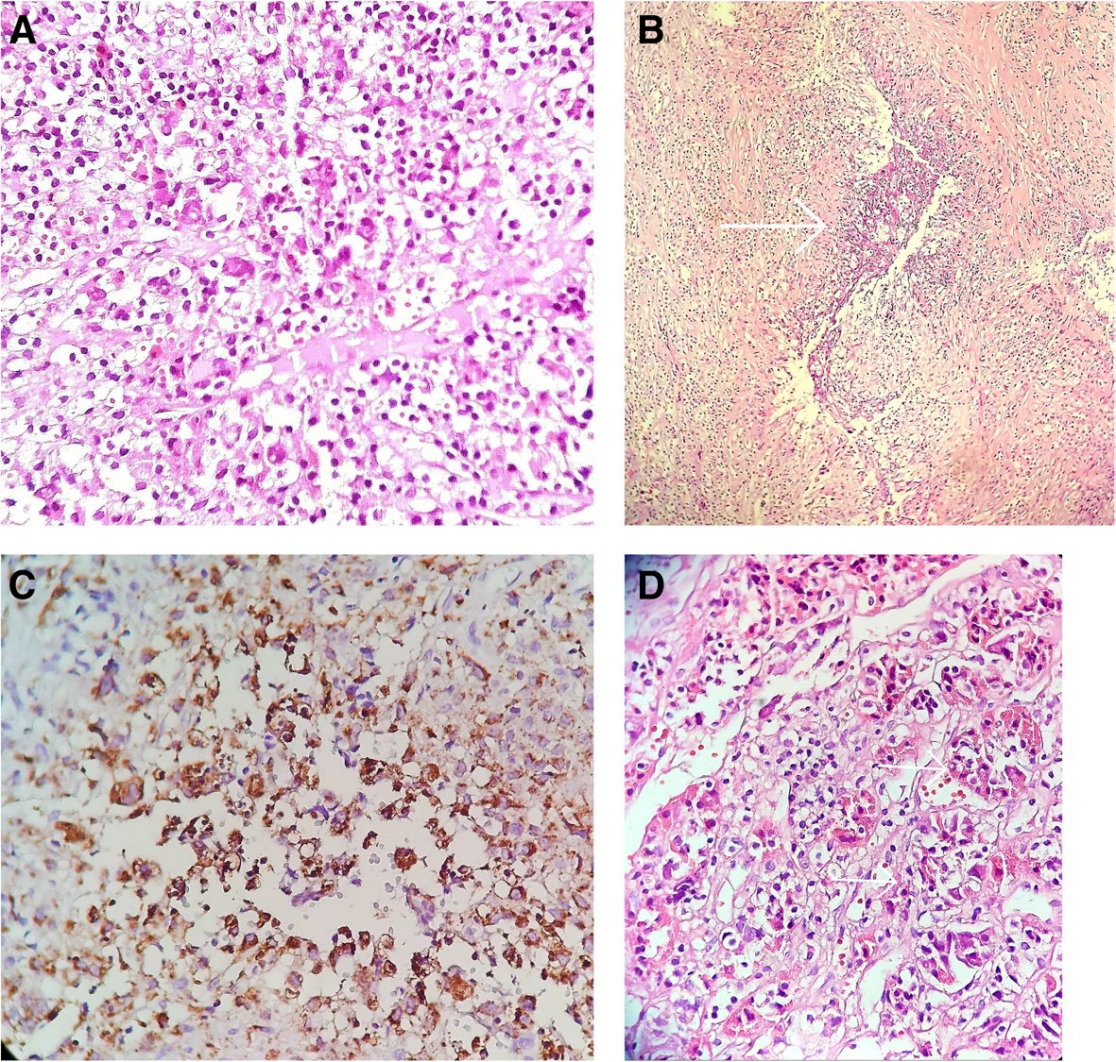
**A** Anterior pituitary glands infiltrated by small mature lymphocytes and histiocytes, H&E stain, × 400. **B** Granulomatous aggregates of epithelioid histiocytes with central neutrophilic microabscess formation (white arrow), H&E stain, × 100. **C** Granulomatous aggregates stained with CD68, × 400. **D** Remnants of inflamed anterior pituitary gland (white arrow), ×400

Histopathologic examination showed an anterior pituitary gland with dense infiltration of lymphocytes, histiocytes, and some granulomas with central neutrophilic microabscess formation. No acid-fast bacilli or fungal elements were noted with Ziehl Neelsen and periodic acid–Schiff (PAS) stains. The final diagnosis was granulomatous hypophysitis with neutrophilic microabscess formation (Fig. 2A, B, C, D).

Excised tissue and pus cultures showed massive growth of *Streptococcus viridans* colonies. All isolates were sensitive to cefotaxim, penicillin, and vancomycin and resistant to clindamycin and erythromycin.

Direct Ziehl–Neelsen staining of pus, histopathology, and a polymerase chain reaction of cerebrospinal fluid and pituitary gland tissue excluded tuberculosis and other mycobacterial agents. Angiotensin-converting enzyme (ACE) levels were normal. Serologic tests for antineutrophil cytoplasmic antibodies (ANCA)-associated vasculitis and syphilis were negative. Repeated blood cultures and echocardiography were normal. Ear and mastoid, and oral cavity evaluations were unremarkable.

### Treatment and follow up

The patient was initially treated with meningeal doses of ceftriaxone (2 g twice daily) and metronidazole (500 mg (mg) four times daily) for a brain abscess with mixed bacterial etiologies presumptive to the oral cavity or paranasal sinus origins. After obtaining the antibiogram and culture results, due to the susceptibility pattern of *S. viridans* and the high probability of mixed etiologies, the above-mentioned regimen was continued for 4 weeks postoperatively, followed by amoxicillin-clavulanate (500/125 mg trice daily) for the total duration of 12 weeks. During the post-operation period (4 weeks later), low levels of cortisol led to replacement therapy in the adrenal axis (Table 2).

**Table 2:** The assessments of pituitary gland function 4 weeks after operation

Test	Measured value	Reference value	Unit
Cortisol (8 A.M)	0.8	10–20	micg/ dl
TSH	0.4	0.45–4.5	mU/ L
T4	6.8	5–12	micg/ dl

*TSH* Thyroid Stimulating Hormone, *T4* Thyroxin

**Fig. 3:**
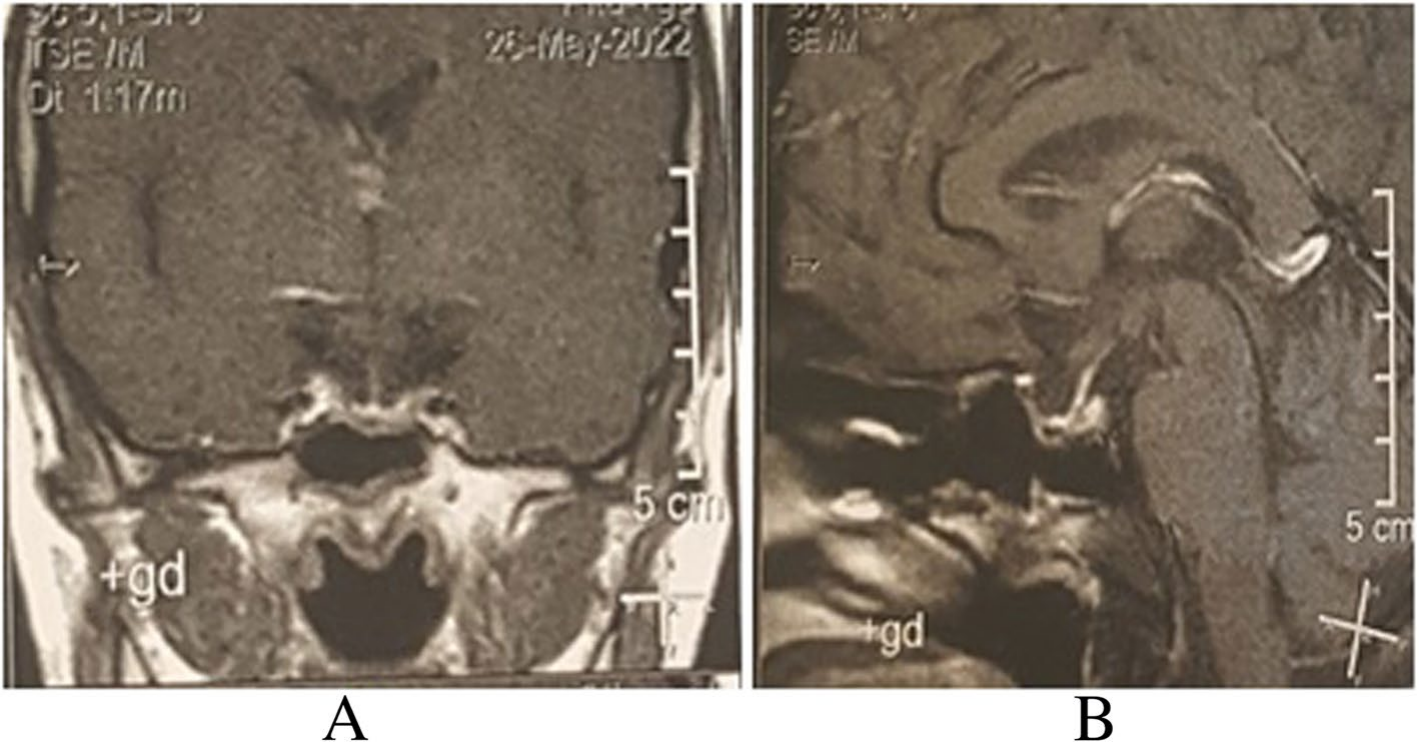
**A**, **B** Sagittal and Coronal post contrast T1-weighted of pituitary gland demonstrate normal pituitary gland remnant without stalk thickening or superior convexity

The patient received appropriate care in the infectious diseases department and was discharged without major complaints after 4 weeks. The patient recovered uneventfully and her menstrual cycle regained its function. Postoperative MRI was normal without any remnant lesion. (Fig. 3A,B).

## Discussion and conclusions

We reported a rare case of granulomatous hypophysitis associated with a pituitary gland abscess caused by S.viridans in a woman who presented with complex manifestations of menstrual disturbance, headache, and vertigo, several months before admission.

The majority of cases of granulomatous hypophysitis were presented with headaches, menstrual changes, nausea, and visual and oculomotor disturbances due to the mass effects of an enlarged pituitary gland, prompting the need for surgical resection of the gland. Moreover, subset of granulomatous hypophysitis cases manifest in a manner that is not due to the effect of mass. Central diabetes insipidus (DI) and hypopituitarism are the most significant clinical manifestations of the disease. Granulomatous hypophysitis may account for some cases of hypopituitarism and central DI where treatment is initiated with no known cause [8]. Due to the difficulty in distinguishing and diagnosing granulomatous hypophysitis from simple adenoma based on the clinical manifestations and radiological findings, histopathological evaluation is required to confirm the diagnosis [8–10].

Granulomatous hypophysitis imaging findings include symmetrical enlargement and homogeneous gadolinium enhancement of the pituitary gland, infundibular thickening, and normal sellar size [8, 11–13], and these also were considered in our case. There was also T1 hypo intense lesion with rim enhancement in the pituitary gland, so the differential diagnosis of our case includes pituitary abscess, tubercoloma, fungal infection, sarcoidosis, granulomatosis with polyangiitis, and Langerhans cell histiocytosis [11–13]. Therefore, adequate histopathological and molecular evaluation of the pituitary gland is the best way to confirm the diagnosis.

Treatment options for primary hypophysitis consist of the administration of corticosteroids, surgical excision, and radiosurgery [14, 15]. Treatment aims to relieve symptoms [16]. Overall, the symptoms were cured in 80% of granulomatous hypophysitis patients [8]. In a literature review of 82 cases, Hunn et al. [8] concluded that there was no difference in symptom resolution, hypopituitarism, and recurrence rates between patients undergoing surgical resection and those undergoing biopsy and steroid treatment. However, when patients were treated with a combination of surgical resection and steroid therapy, outcomes were worse than those treated with surgical resection alone. There is only a case report of granulomatous hypophysitis reporting that prednisolone was ineffective [17]. On the other hand, recent case reports have documented a reduction in neurological symptoms following corticosteroid use, and many studies have advocated corticosteroid treatment over an excisional strategy alone [15, 16, 18].

A pituitary abscess is a rare and life-threatening disease [6], accounting for less than 1% of all pituitary lesions. The exact etiology and pathogen remain unclear in a significant percentage of cases. Primary pituitary abscesses may arise in the normal pituitary gland, either by hematogenous seeding or by direct extension of adjacent infection through the sphenoid sinus [19], or as a complication of cavernous sinus thrombosis [20]. A secondary pituitary abscess occurs in a gland that has a pre-existing lesion. Pituitary adenoma, Rathke’s cleft cyst, craniopharyngioma, lymphoma [7], Immunocompromised conditions, and previous pituitary surgery or irradiation are other risk factors that may lead to a pituitary abscess [20, 21].

In a study of 24 cases of a pituitary abscess, 41.7% had a history of pituitary surgery or sphenoid sinus disease, 19.7% of these patients had an obvious source of sepsis or bacteremia, 12.5% had a sensation of CSF leakage and rhinorrhea, 8.3% had a history of brain radiation, and 4.2% had a history of sphenoid sinusitis [22]. Nevertheless, the etiology of the pituitary abscess has remained unrecognized in 60% of cases [23]. Moreover, there is no evidence of any association between granulomatous hypophysitis and pituitary abscess. Focal destruction of adjacent paranasal sinuses and direct inoculation, hematogenous seeding on inflamed gland tissue, and increased risk of superinfection due to prolonged corticosteroid ingestion for the hormonal disorder could be possible presumptions. However, these possible etiologies were not compatible with our patient.

The patient’s medical history was further examined to rule out possible secondary causes of the pituitary abscess. The patient had no history of surgery, medical history, sinusitis, and radiation exposure. Radiographic findings also showed no evidence of cavernous sinus thrombosis or sinusitis. Our study failed to find a possible cause of the pituitary abscess.

## Data Availability

Not applicable.

## Abbreviations

MRI: magnetic resonance imaging
TSS: transsphenoidal surgery
PAS: periodic acid–Schiff
ACE: Angiotensin-converting enzyme
ANCA: antineutrophil cytoplasmic antibodies
